# Small bowel obstruction complicating an *Ascaris lumbricoides* infestation in a 4-year-old male: a case report 

**DOI:** 10.1186/s13256-019-2103-y

**Published:** 2019-05-24

**Authors:** Clarence Mvalo Mbanga, Kingsley S. Ombaku, Karl Njuwa Fai, Valirie Ndip Agbor

**Affiliations:** 1 Mankon Sub Divisional Hospital, Bamenda, Cameroon; 2 Wum District Hospital, Wum, Cameroon; 3 Ibal Sub-Divisional Hospital, Oku, Cameroon; 4 Department of Clinical Research, Health Education and Research Organization (HERO), Buea, Cameroon

**Keywords:** Ascariasis, Soil-transmitted helminthiasis, Intestinal obstruction, Cameroon

## Abstract

**Background:**

Ascariasis is the leading helminthic infection worldwide, with its peak prevalence noted in children aged 2–10 years. Although mainly asymptomatic, chronic and heavy infestation could lead to severe complications such as malnutrition, poor physical and cognitive development, as well as intestinal obstruction. We report the case of a 4-year-old boy with intestinal obstruction due to *Ascaris lumbricoides* infestation and discuss its public health significance.

**Case presentation:**

A 4-year-old Black African boy from the Menchum Division in the Northwest Region of Cameroon, with no history of deworming since birth, presented with a 3-day history of generalized abdominal pains, vomiting and obstipation, and abdominal distention evolving over a period of 6 months. Clinical and paraclinical findings were in favor of a subacute intestinal occlusion associated with an electrolyte imbalance. An exploratory laparotomy was done after correction of the electrolyte imbalance. Perioperative findings revealed a dilated small bowel obstructed by bundles of live worms. An enterotomy of 2 cm in length was done, and the bundles of *Ascaris lumbricoides* worms extracted manually and by milking through the stoma. His postoperative period was unremarkable, and he was discharged on postoperative day 7. He and his entire household were dewormed with a single dose of mebendazole 500 mg administered orally. A follow-up visit 1 week after discharge revealed a healed abdominal wound and normal bowel functions.

**Conclusion:**

Despite considerable progress made on the control of soil-transmitted helminthiasis in Cameroon, the program faces a number of bottlenecks. Funding is inadequate, making data acquisition and hence remapping of high-risk zones difficult. Accessibility to enclaved zones where most high-risk children live is difficult, while community sensitization on soil-transmitted helminthiasis and proper education on the right environmental hygienic practices are lacking. All these challenges once addressed could go a long way to help achieve recently set sustainable development goals.

## Background

Ascariasis remains the leading helminthic infection worldwide, disproportionally affecting children in tropical countries and low-income and middle-income countries [1]. Ascariasis affects approximately 0.8 billion people globally, with the highest prevalence noted among children aged 2–10 years [1]. Although mainly asymptomatic, symptomatic ascariasis could manifest as pneumonitis, hepatobiliary or pancreatic damage, growth retardation, intestinal obstruction (IO), or peritonitis [2].

Ascariasis is a common cause of IO in children [3–5], with peak age between 2 and 10 years [5–7]. Treatment options are variable and generally entail external bowel resection and end-to-end anastomosis, enterotomy and milking out of worms, or manual exposition and advancement of the mass of writhing worms toward the colon [6, 7]. Here, we report the case of a 4-year-old boy with mechanical IO due to *Ascaris lumbricoides* (*A. lumbricoides*) successfully treated by enterotomy and milking out of the worm bundle. In addition, we highlight how a single case of IO due to *A*. *lumbricoides* could reflect loopholes in public health strategies put in place to control and eradicate the condition, and discuss possible solutions. This is particularly important in the context of achieving the ambitious target of the sustainable development goal (SDG) 3.3 which seeks to eradicate neglected tropical diseases by 2030.

## Case presentation

A 4-year-old Black African boy (from a rural locality in the Menchum Division of the Northwest Region of Cameroon), with no remarkable past medical and family history, consulted our emergency unit for a 3-day history of generalized abdominal pains, vomiting, and obstipation. We also noted an abdominal distention which his mother ascertained to have been evolving for 6 months prior to consultation at our health facility. In addition, the child had never been dewormed since birth according to the mother.

A physical examination revealed a conscious but asthenic patient with signs of malnutrition and some dehydration. His conjunctivae were pinkish and sclerae were anicteric. His abdomen was distended, soft but mildly tender, mobile with respiration, and dull on percussion. There was no palpable abdominal mass or shifting dullness. Bowel sounds were hyperactive, and the rectum was void of fecal material on digital rectal examination. Initial laboratory investigations revealed hypokalemia and hyponatremia. A full blood count was normal. A plain abdominal X-ray revealed discrete air-fluid levels. Based on the aforementioned clinical and paraclinical findings, a diagnosis of IO was arrived at. Further exploration of the cause of the obstruction was inaccessible mostly because our patient’s family could not afford the cost, and the nearest referral facility capable of performing these tests was approximately 76 km away, on poorly motorable and hilly roads. Taking these circumstances and the deteriorating clinical picture of our patient into account, we decided to do an exploratory laparotomy after receiving a verbal and signed consent from our patient’s carer.

He was admitted, rehydrated with 2 L of Ringer’s lactate and 1 L of glucose 5% per m^2^/day for 3 days, and intravenously administered paracetamol 15 mg/kg per 6 hours, ceftriaxone 50 mg/kg per day, metronidazole 15 mg/kg per 8 hours, and gentamycin 5 mg/kg per day. His legal guardian was immediately counselled on the need for a laparotomy and a signed informed consent was obtained after which an anesthetic consult was sought. He was operated on the third day of hospitalization after correction of the associated electrolyte imbalance.

**Fig. 1:**
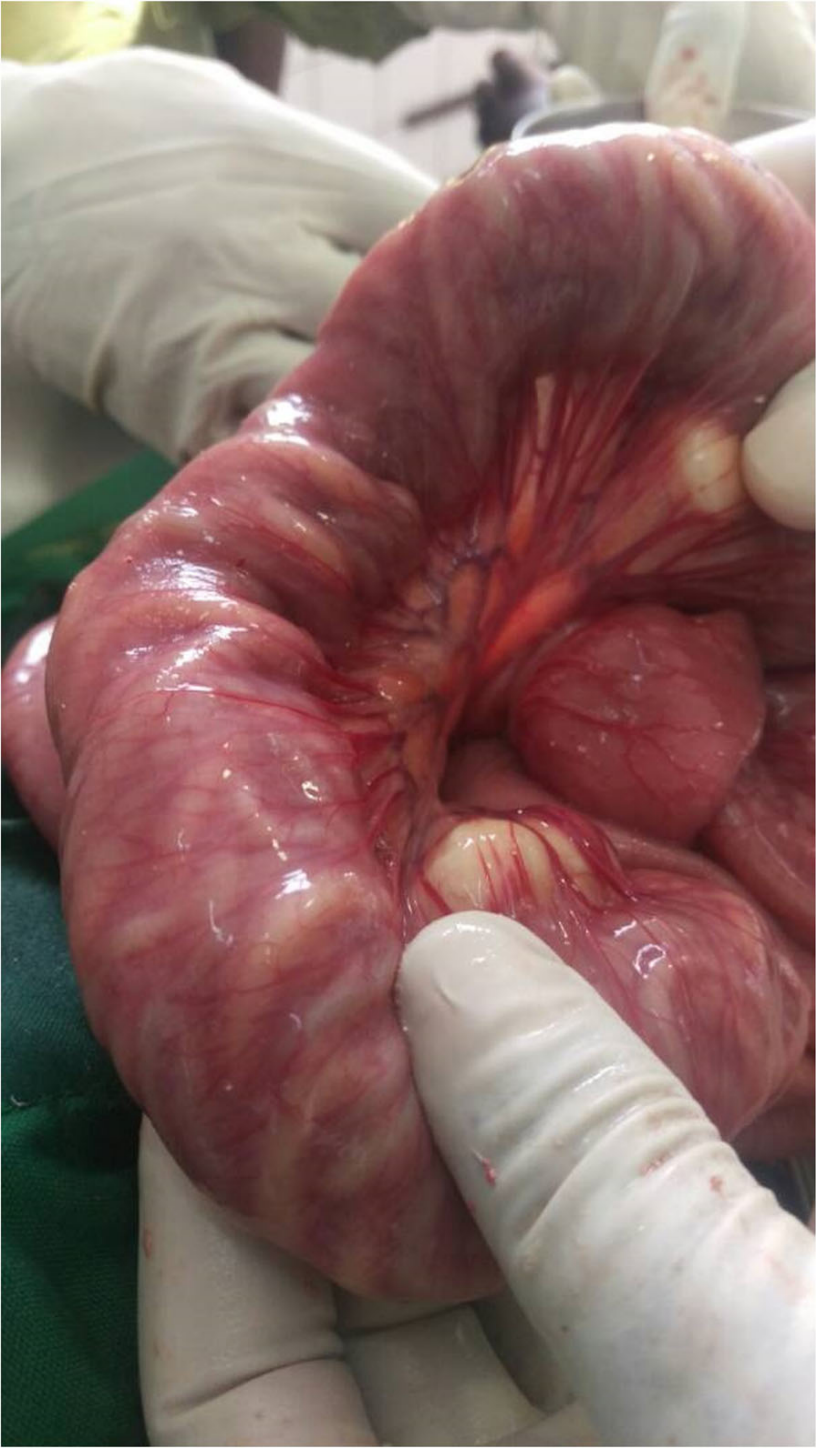
Distended small bowel obstructed by bundles of *Ascaris lumbricoides* worms

**Fig. 2:**
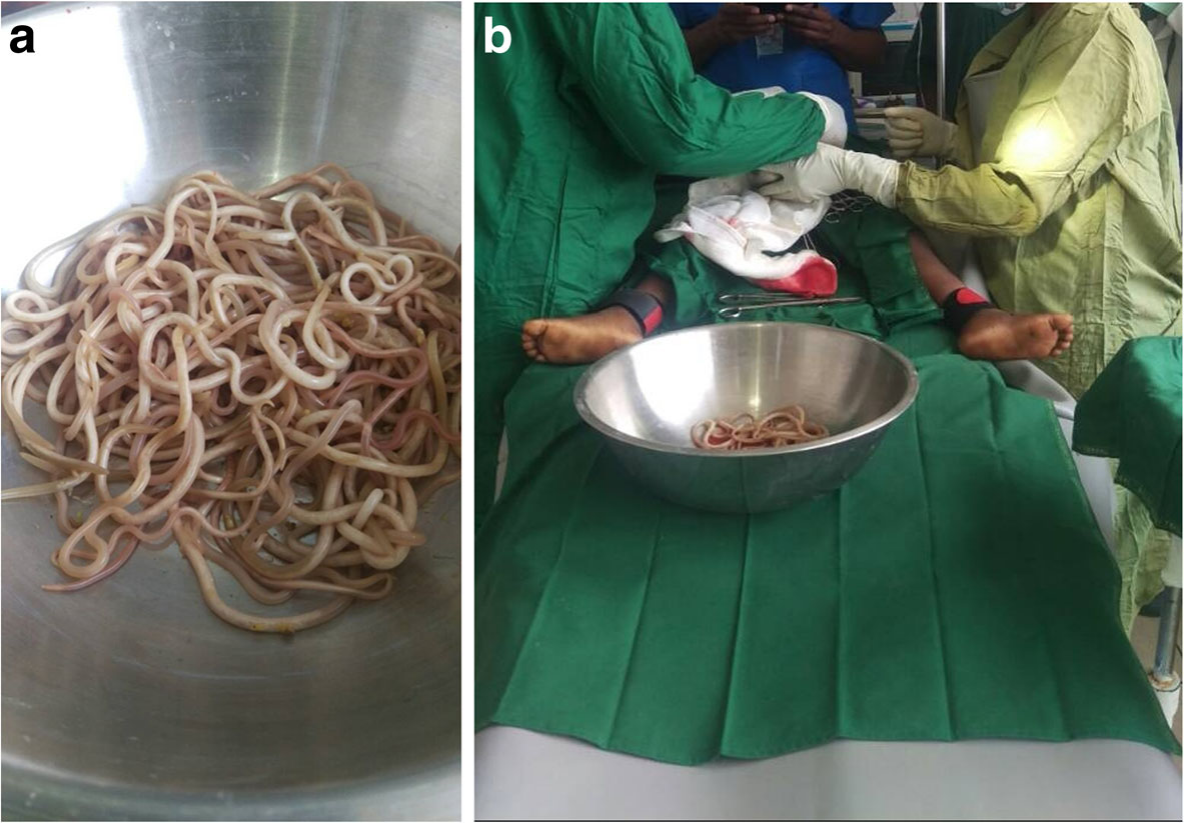
**a**, **b** Bundle of *Ascaris lumbricoides* worms extracted after enterotomy

The surgical approach consisted of the traditional midline incision. Perioperative findings revealed a dilated small bowel obstructed by bundles of live worms (Fig. 1). An enterotomy of 2 cm in length which exposed the bundles of *A. lumbricoides* was done, followed by manual extraction and milking of the worms through the stoma (Fig. 2 a, b). Postoperative management involved intravenously administered fluids with Ringer’s lactate, and intravenously administered paracetamol 15 mg/kg per 6 hours, ceftriaxone 50 mg/kg per day, metronidazole 15 mg/kg per 8 hours, and gentamycin 5 mg/kg per day. Progressive oral sips were started 8 hours after surgery and semi-solid food was introduced from postoperative day 3. Evolution was favorable with full restoration of bowel function on postoperative day 3. Our patient was discharged on postoperative day 7 with no fresh complaints. He and his entire household were dewormed with a single dose of mebendazole 500 mg administered orally. A follow-up visit 1 week after discharge revealed a healed abdominal wound and normal bowel functions.

## Discussion

Ascariasis is a round intestinal worm infection transmitted by accidental ingestion of eggs present in human feces that contaminate food, soil, and water in areas of poor hygiene [8]. Like other neglected tropical diseases, it is indicative of a poor socioeconomic status, hygiene, and sanitation in a given population. *A*. *lumbricoides* shares a similar mode of transmission with the whipworm (*Trichuris trichiura*) and hookworms (*Ancylostoma duodenale* and *Necator americanus*) and they are collectively referred to as soil-transmitted helminthiasis (STH) [8]. STH are the world’s most common parasitic infection, affecting an estimated two billion people worldwide, most of whom live in developing countries [8].

The morbidity and symptomatology of ascariasis is linked to the worm burden in the small intestine where they mature, copulate, and produce eggs [8]. Our patient was 4-years old and reported to have never been dewormed since birth. This most probably led to a high worm burden and culminated in IO. This highlights the importance of regular deworming (biannually or at least annually) to clear off adult worms and their eggs from the bowel, in order to reduce worm burden and prevent the occurrence of life-threatening chronic complications such as IO among others.

Heavy and chronic infection could lead to complications such as malnutrition, poor physical growth, and poor memory and cognition, especially among school-age children [9] because the adult worms live in the small bowel and feed off its content. Our patient presented with signs of malnutrition. This could be explained by the probable chronic evolution of his ascariasis, with gradual increase in the worm burden which in turn fed off the micronutrients in his small bowel, eventually leading to malnutrition.

Ascariasis is the leading cause of disability-adjusted life years (DALYs) lost among the various STH with an estimated 1094.67 thousand years [10]. However, *A*. *lumbricoides* is still termed the “neglected parasite” [11]; probably because ascariasis is not linked to high levels of mortality, but rather has most of its burden associated with its morbidity (which usually ensues after several years of infestation). As a result, it is given relatively less attention than other infectious diseases associated with higher mortality rates. Sub-Saharan Africa accounts for 16% of an estimated 1.01 billion school-age children regarded to be at risk of infection by one or more STH species [1]. Cameroon was among the countries with the most elevated transmission rates of *A*. *lumbricoides* in sub-Saharan Africa in 2010 [1], highlighting the local public health burden of the condition. In 2007, the Ministry of Public Health set in motion a national deworming campaign for all school-age and pre-school-age children. Twice yearly, national health weeks are organized to deworm, via a school-based and door-to-door approach, children of age 1 to 5 years using mebendazole [12]. This has proven to be effective in the control of STH, with a review of the program in 2013 revealing a considerable decrease in the prevalence of STH in Cameroon [13]. However, re-infection rates of dewormed children remains unacceptably high, hampering diseases control and/or eradication [14]. In addition, the transmission and prevalence of STH are known to change with time and intervention [15]. Consequently, it is imperative that re-mapping of high-risk zones be done so that the program can be tailored to constantly target children at greatest risk of STH infection.

The STH control program in Cameroon faces a number of bottlenecks. For example, community health education, with an emphasis on environmental hygiene and sanitation, is only given particular attention during national campaign or local campaign days. Also, community involvement and social mobilization during campaigns is poor owing to insufficient payment of community drug distributors (CDDs) and health area supervisors. This payment is usually not encouraging and/or delayed, leading to demotivation of these actors and consequent attrition [16, 17]. In addition, the deworming program might not be exhaustive, as out-of-school children and those living in rural and enclaved zones are likely to be missed. The child presented in our case report was from a rural locality in Menchum Division of the Northwest Region of Cameroon, which is a hard-to-reach area owing to poorly motorable and hilly roads. With little motivation, these areas are hardly covered by CDDs and program supervisors.

Some additions could enhance the success of deworming campaigns. The community needs to be sensitized on the importance of regular deworming and healthy practices such as: boiling water prior to drinking; proper hand washing with water and soap before handling food; and proper washing and cooking of vegetables before their consumption. Discouraging practices such as the use of human feces as manure in farms, while encouraging the use of toilets (avoiding open defecation) and development of proper animal (particularly pig) sewage disposal systems could equally help prevent transmission of STH [14, 18]. Health education is crucial in curbing re-infection rates and should therefore constitute an integral part of campaigns. This environmental and health education needs to be made a routine point of discussion with the children at school, and with the general population at traditional gatherings, via local radio stations and even in churches; and not only on vaccination days. Training and motivating health professionals or community health workers could improve routine health education of the local population. Strategies such as farm and market approaches could be introduced to catch up with out-of-school children missed by the currently used school-based approach. Efficiently mobilizing the community to support CDDs either financially or materially will improve motivation and, hence, treatment coverage. All of these changes if properly implemented could go a long way to control and/or eradicate STH in Cameroon and help achieve the SDG 3.3 [19].

## Conclusion

*A*. *lumbricoides* is a public health concern in Cameroon given its association with significant morbidity especially in school-age children. Considerable progress has been made on the control of STH in Cameroon. However, with the high reinfection rate, efforts need to be channeled on improving disease control at local levels through community sensitization on STH, motivation of local actors, and proper education on the right environmental hygiene practices.

## Abbreviations

CDDs: Community drug distributors
IO: Intestinal obstruction
SDGs: Sustainable development goals
STH: Soil-transmitted helminthiasis

## References

[1] Pullan RL, Smith JL, Jasrasaria R, Brooker SJ. Global numbers of infection and disease burden of soil transmitted helminth infections in 2010. Parasit Vectors. 2014;7:37. 10.1186/1756-3305-7-37 PMC3905661 24447578

[2] Martin E, William T, Harry V. Zoonoses; Recognition, Control, and Prevention. Iowa State: Blackwell Publication Company Iowa State Ames USA; 2000.

[3] Shiekh KA, Baba AA, Ahmad SM, Shera AH, Patniak R, Sherwani AY. Mechanical small bowel obstruction in children at a tertiary care center in Kashmir. Afr J Paediatr Surg. 2010;7(2):81–5. 10.4103/0189-6725.62852 20431216

[4] Mir M, Bucch M, Younus U, Sheikh GM, Bali B. Clinical study of mechanical small-bowel obstruction in children in Kashmir. Internet J Surg. 2012;28:2.

[5] Ooko PB, Wambua P, Oloo M, Odera A, Topazian HM, White R. The Spectrum of Paediatric Intestinal Obstruction in Kenya. Pan Afr Med J. 2016;24:43. 10.11604/pamj.2016.24.43.6256 PMC5012794 27642384

[6] Upadhyaya V, Gangopadhyay A, Pandey A, Gupta D, Upadhyaya A. Round worm intestinal obstruction: a single center study. Internet J Surg. 2007;12:2.

[7] Wani I, Rather M, Naikoo G, Amin A, Mushtaq S, Nazir M. Intestinal ascariasis in children. World J Surg. 2010;34(3):963–8. 10.1007/s00268-010-0450-3 20143068

[8] World Health Organization. WHO. Soil-transmitted helminth infections [Internet]. [cited 2017 Jan 28]. Available from: http://www.who.int/mediacentre/factsheets/fs366/en/.

[9] Bethony J, Brooker S, Albonico M, Geiger SM, Loukas A, Diemert D, *et al.* Soil-transmitted helminth infections: ascariasis, trichuriasis, and hookworm. Lancet. 2006;367:1521–32. 10.1016/S0140-6736(06)68653-4 16679166

[10] World Health Organization. WHO. Health Statistics and Information Systems. Estimates for 2000–2015. Disease Burden [Internet]. [cited 2018 Jan 28]. Available from: http://www.who.int/healthinfo/global_burden_disease/estimates/en/index2.html.

[11] Holland C. Ascaris: The Neglected Parasite. 1st ed. San Diego: Academic Press; 2013.

[12] Tchuem Tchuenté LA, N’Goran EK. Schistosomiasis and soil-transmitted helminthiasis control in Cameroon and Côte d’Ivoire: implementing control on a limited budget. Parasitology. 2009;136:1736–45. 10.1017/S0031182009005988 19490723

[13] Tchuem Tchuente LA, Dongmo NC, Ngassam P, Kenfack CM, Feussom GN, *et al.* Mapping of schistosomiasis and soil-transmitted helminthiasis in the regions of Littoral, North-West, South and South-West Cameroon and recommendations for treatment. BMC Infect Dis. 2013;13:602. 10.1186/1471-2334-13-602 PMC3878270 24365046

[14] Hadush A, Pal M. Ascariasis: Public Health Importance and its Status in Ethiopia. Air Water Borne Dis. 2016;5:1.

[15] Truscott J, Hollingsworth TD, Anderson R. Modelling the interruption of the transmission of soil-transmitted helminths by repeated mass chemotherapy of school-age children. PLoS Negl Trop Dis. 2014;8(12):e3323. 10.1371/journal.pntd.0003323 PMC4256169 25474477

[16] Tchuem Tchuente LA. Control of soil-transmitted helminths in sub-Saharan Africa: Diagnosis, drug efficacy concerns and challenges. Acta Trop. 2011;120(Suppl 1):S4–S11. 10.1016/j.actatropica.2010.07.001 20654570

[17] Our Cameroon Partners-COUNTDOWN-Calling time on Neglected Tropical Diseases [Internet]. [cited 2018 Jan 29]. Available from: http://countdowncameroon.org/.

[18] Asaolu SO, Ofoezie IE. The role of health education and sanitation in the control of helminth infections. Acta Trop. 2003;86(2–3):283–94. 10.1016/s0001-706x(03)00060-3 12745145

[19] Health-United Nations Sustainable Development Goals [Internet]. [cited 2018 Jan 31]. Available from: http://www.un.org/sustainabledevelopment/health/.

